# Personalized Behavioral Feedback for Online Gamblers: A Real World Empirical Study

**DOI:** 10.3389/fpsyg.2016.01875

**Published:** 2016-11-28

**Authors:** Michael M. Auer, Mark D. Griffiths

**Affiliations:** ^1^neccton LtdLienz, Austria; ^2^Psychology, Nottingham Trent UniversityNottingham, UK

**Keywords:** online gambling, responsible gambling, problem gambling, human–computer interaction, behavioral feedback, persuasive communication

## Abstract

Responsible gambling tools (e.g., limit-setting tools, pop-up messages, and personalized feedback) have become increasingly popular as a way of facilitating players to gamble in a more responsible manner. However, relatively few studies have evaluated whether such tools actually work. The present study examined whether the use of three types of information (i.e., personalized feedback, normative feedback, and/or a recommendation) could enable players to gamble more responsibly as assessed using three measures of gambling behavior, i.e., theoretical loss (TL), amount of money wagered, and gross gaming revenue (GGR) (i.e., net win/loss). By manipulating the three forms of information, data from six different groups of players were analyzed. The participant sample drawn from the population were those that had played at least one game for money on the *Norsk Tipping* online platform (*Instaspill*) during April 2015. A total of 17,452 players were randomly selected from 69,631 players that fulfilled the selection criteria. Of these, 5,528 players participated in the experiment. Gambling activity among the control group (who received no personalized feedback, normative feedback or no recommendation) was also compared with the other five groups that received information of some kind (personalized feedback, normative feedback and/or a recommendation). Compared to the control group, all groups that received some kind of messaging significantly reduced their gambling behavior as assessed by TL, amount of money wagered, and GGR. The results support the hypothesis that personalized behavioral feedback can enable behavioral change in gambling but that normative feedback does not appear change behavior significantly more than personalized feedback.

## Introduction

Gambling is a popular activity in many cultures. Surveys have reported that most people gamble but do so infrequently (e.g., Wardle et al., 2007). National surveys have also concluded that most people have engaged in gambling at some point during in their lives (Orford et al., 2003; Meyer et al., 2009). In Great Britain, the majority of the population (over two-thirds) engaged in at least one type of gambling in the previous 12 months (Wardle et al., 2012). This included oﬄine and online gambling. A recent review by Gainsbury (2015) on internet gambling reported that in jurisdictions that have carried out studies, online gambling prevalence rates are still relatively low (8–16%). However, as internet gambling is accessible 24 h a day, potentially negative psychosocial impacts are an almost inevitable consequence for a small minority of individuals. This is because various structural and situational characteristics (e.g., accessibility, affordability, and anonymity) may increase the risk of developing a gambling problem among vulnerable and susceptible individuals (Griffiths, 2003; McCormack and Griffiths, 2013). Consequently, players need to be educated about how to gamble more responsibly and vulnerable groups need to be protected.

### Responsible Gambling and Information Giving

Responsible gambling tools (e.g., limit-setting tools, pop-up messages, and personalized feedback) have become increasingly popular as a way of facilitating players to gamble in a more responsible manner (Griffiths et al., 2009; Auer and Griffiths, 2013). They are also given information about common misconceptions and erroneous perceptions concerning games of chance as these have been found to be important factors in the acquisition, development, and maintenance of problematic gambling (e.g., Gaboury and Ladouceur, 1989; Griffiths, 1994; McCusker and Gettings, 1997; Parke et al., 2007; Wohl et al., 2010). However, empirical evaluations demonstrating that providing gamblers with such information in an attempt to correct or change erroneous beliefs and misperceptions have been variable. For instance, some studies have supported the use of providing information in helping individuals gamble more responsibly (e.g., Dixon, 2000; Ladouceur and Sevigny, 2003), while other studies have reported no significant association between providing information and gambling responsibly (e.g., Hing, 2003; Focal Research, 2004; Williams and Connolly, 2006).

### Responsible Gambling and Correcting Erroneous Beliefs

Some studies have successfully utilized educational programs as a way of correcting erroneous beliefs about gambling (e.g., Wulfert et al., 2006; Wohl et al., 2010). For example, animation-based educational videos have been developed that educate gamblers about how slot machines work (Wohl et al., 2010). After watching the animated video, gamblers said they intended to use strategies to (i) stay within their limits, and (ii) reduce the number of times they would exceed their limits. The same study also demonstrated that animated videos may be an effective tool for increasing the likelihood of gamblers setting financial spending limits.

### Responsible Gambling Messaging and How it Is Presented

Research has also shown that the way information is presented can significantly influence behavior and thoughts. Several studies have investigated the effects of interactive vs. static pop-up messages during gambling sessions. Static messages do not appear to be particularly effective, whereas interactive pop-up messages and animated information have been shown to change irrational belief patterns and subsequent gambling behavior (e.g., Schellink and Schrans, 2002; Ladouceur and Sevigny, 2003; Cloutier et al., 2006; Monaghan and Blaszczynski, 2007, 2010a; Monaghan et al., 2009).

It has also been recommended that warning signs containing information should utilize skills that facilitate self-regulation and self-appraisal rather than just simply providing information (Monaghan and Blaszczynski, 2010b). For instance, an experimental study on slot machine players by Monaghan and Blaszczynski (2010a) demonstrated pop-up messages that contained self-appraisal messages resulted in more self-reported thoughts and behavior while gambling compared to those that do not.

### Responsible Gambling and Pop-Up Messaging

A study by Stewart and Wohl (2013) found that individuals were significantly more likely to stick to monetary limits while gambling if they received a pop-up reminder about monetary limits compared to those that did not. In a similar study, Wohl et al. (2013) examined the efficacy of two different responsible gambling tools (a pop-up message and an educational animated video) in relation to money limit adherence while gambling on a slot machine (*n* = 72). The authors reported that both tools were effective in helping gamblers keep within their predetermined financial spending limits. Munoz et al. (2013) conducted a study to examine whether graphic warning signs had greater efficacy than text-only warning signs. They reported that the graphic warnings were more successful than text warnings in getting gamblers to comply with the advice given, and more successful in getting participants to change their attitudes concerning gambling. While all of these studies have provided empirical support for the use of responsible gambling tools, they are limited by the small sample sizes and the lack of ecological validity (i.e., the studies were carried out in a laboratory situation).

More recently, a number of studies have been carried out in real world settings using real gamblers in real time. For instance, Auer et al. (2014) investigated the effect of a pop-up message that appeared after 1,000 consecutive online slot machine games had been played by individuals during a single gambling session. The study analyzed 800,000 gambling sessions (400,000 sessions before the pop-up had been introduced and 200,000 after the pop-up had been introduced comprising around 50,000 online gamblers). The study found that the pop-up message had a limited effect on a small percentage of players. More specifically, prior to the pop-up message being introduced, five gamblers ceased playing after 1,000 consecutive spins of the online slot machine within a single playing session (out of approximately 10,000 playing sessions). Following the introduction of the pop-up message, 45 gamblers ceased playing after 1,000 consecutive spins (i.e., a ninefold increase in session cessations). In the latter case, the number of gamblers ceasing play was less than 1% of the gamblers who played 1,000 games consecutively.

In a follow-up study, Auer and Griffiths (2015a) argued that the original pop-up message was very basic and that re-designing the message using normative feedback and self-appraisal feedback may increase the efficacy of gamblers ceasing play. As in the previous study, the new enhanced pop-up message that appeared within a single session after a gambler had played 1,000 consecutive slot games. In the follow-up study, Auer and Griffiths (2015a) examined 1.6 million playing sessions comprising two conditions [i.e., simple pop-up message (800,000 slot machine sessions) vs. an enhanced pop-up message (800,000 slot machine sessions)] with approximately 70,000 online gamblers. The study found that the message with enhanced content more than doubled the number of players who ceased playing (1.39% who received the enhanced pop-up compared to 0.67% who received the simple pop-up). However, as in Auer et al.’s (2014) previous study, the enhanced pop-up only influenced a small number of gamblers to cease playing after a long continuous playing session. At present, these two research studies (i.e., Auer et al., 2014; Auer and Griffiths, 2015a) are the only ones to examine the efficacy of pop-up messaging in a real world online gambling environment comprising actual online gamblers.

### Responsible Gambling and Personalized Feedback via Behavioral Tracking Tools

Personalized feedback which informs players about their past behavior and incorporates a longer time period than just the current session has only been empirically researched in one real-world study to date. Auer and Griffiths (2015b) studied the behavior of 1,015 online gamblers in connection with their voluntary use of a responsible gaming behavioral tracking tool compared with 15,216 matched control group gamblers (that had not used the behavioral tracking tool) on the basis of age, gender, playing duration, and theoretical loss (TL) [i.e., the amount of money wagered multiplied by the payout percentage of a specific game played (Auer et al., 2012; Auer and Griffiths, 2014)]. The results showed that online gamblers receiving personalized feedback spent significantly less money and time gambling in comparison to those that did not receive personalized feedback (i.e., the matched controls). However, as gamblers who had used the behavioral tracking tool had volunteered to use it and had not been randomly assigned, this meant the effect might not only be due to the feedback but also to other factors not controllable by the researchers (for instance, those signing up to use the tool may have been more responsible gamblers to begin with).

Forsström et al. (2016) carried out a study on the use of the behavioral tracking tool *PlayScan.* The data from a total of 9,528 players who voluntarily used the system were analyzed. They found that the initial usage of the tool was high, but that repeated usage was low. Two groups of users (i.e., ‘self-testers’ and ‘multi-function users’) utilized the tool to a much greater extent than other groups. However, the study did not analyze changes in behavior as a consequence of using the tool. Wood and Wohl (2015) obtained data from 779 *Svenska Spel* online players who received behavioral feedback using *PlayScan*. Feedback to players took the form of a ‘traffic-light’ risk rating that was created via a proprietary algorithm (red = problematic gambling, yellow = at-risk gambling, and green = no gambling issues). In addition, expenditure data (i.e., amounts deposited and gambled) were collected at three time points (i) the week of *PlayScan* enrollment, (ii) the week following *PlayScan* enrollment, and 24 weeks after *PlayScan* enrollment. The findings indicated that those players at-risk (yellow gamblers) who used *PlayScan* significantly reduced the amounts of money both deposited and gambled compared to those who did not use *PlayScan*. This effect was also found the week following *PlayScan* enrollment as well as the 24-week mark. Overall, the authors concluded that informing at-risk gamblers about their gambling behavior appeared to have a desired impact on their subsequent monetary spending.

### Personalized Feedback and Self-Efficacy

The focus of social cognitive theory is self-efficacy and is learned by observing other individuals’ behavior (Bandura, 2001). Self-efficacy primarily concerns how capable an individual feels about performing a behavior and is at the heart of the health communication literature including the Health Belief Model (Maiman and Becker, 1974; Janz and Becker, 1984), Theory of Planned Behavior (Ajzen, 1985), Protection Motivation Theory (Rogers, 1983), and the Extended Parallel Process Model (Witte, 1992). All these theoretical perspectives assert that high levels of self-efficacy are highly likely to enable behavioral change. Consequently, it is important that to enable behavioral change, messages must include components of self-efficacy (i.e., belief that the person can carry out an action) and response efficacy (i.e., belief that the recommended action will lead to a desired outcome for the person; Witte et al., 2001; Perloff, 2008).

Another method of attempting to enable behavioral change in gambling is normative feedback. Studies researching smoking (Van den Putte et al., 2009), condom use (Yzer et al., 2000), and marijuana consumption (Yzer et al., 2007) have shown normative beliefs can play an important role in behavioral change. In a study of American college student gambling, Celio and Lisman (2014) demonstrated that personalized normative feedback decreased other students’ perceptions of gambling and lowered risk-taking performance on two analog measures of gambling. They concluded that a standalone personalized normative feedback intervention may modify gambling behavior among college students. Miller and Rollnick (1991) have also emphasized that normative feedback is important in facilitating behavioral change in the use of motivational interviewing.

Outside of the gambling studies field, personalized behavioral feedback has been used to change other potentially addictive behaviors (e.g., cigarette smoking). Using a combination of both motivational interviewing and feedback from ultrasound was found as effective for reducing cigarette smoking among pregnant women (Stotts et al., 2009). Another study effectively delivered a smoking-cessation intervention via wireless text messages to college students using integrated internet/mobile phone technology (Obermayer et al., 2004). Another area where behavioral feedback has been investigated is in the area of sports and fitness. Buttussi et al. (2006) investigated the use of mobile phone guides in fitness activities using a Mobile Personal Trainer (MOPET) application. The mobile app gave verbal navigation assistance and also used a 3D-animated motivator. Evaluation of the results supported the use of mobile apps and embodied virtual trainers in outdoor fitness applications.

Many of the aforementioned approaches enabling behavioral change utilize the ‘stages of change’ model (Prochaska and DiClemente, 1983; Prochaska and Prochaska, 1991) and motivational interviewing (Miller and Rollnick, 1991). Based on these theoretical approaches, the present authors believe that to change an individual’s gambling behavior, player feedback (utilizing behavioral tracking data) has to incorporate the stages of change model and be presented in a motivational way. More specifically, this means providing informational feedback to gamblers in a non-judgmental format along with normative information so that they can evaluate their gambling behavior compared to others like themselves. Transparent and non-judgmental feedback is important and has been emphasized by studies elsewhere. For instance, a study examining alcohol drinking by Lapham et al. (2012) advocated that feedback should be transparent and tailored to the individual (e.g., the extent to which individuals exceeded daily or weekly alcohol limits). This is because their participants explicitly wanted to know how they came to be specifically assigned to their alcohol drinking risk category.

### Human–Computer Interaction and Persuasive System Design in Responsible Gambling

Given that the primary aim of gambling pre-commitment tools is to enable behavioral change, it is only recently that designs of such tools have utilized the principles of human–computer interaction (HCI) and persuasive system design (PSD). A recent study found that a monetary limit pop-up tool inspired by PSD and HCI principles was much more effective than tools not incorporating such principles (Wohl et al., 2014). As a research field, HCI examines the interaction of individuals with technology and attempts to facilitate usability and uptake. Persuasive Technology has been defined as interactive computing systems that attempt to change people’s attitudes and behaviors (Fogg, 2003). Apart from user-feedback, HCI principles relevant for the design of pre-commitment measures are an aesthetic visual design, the incorporation of system-status updates, a sense of control over functionality, and the use of simple language (Hewett et al., 1992; Shneiderman et al., 2009; Preece et al., 2011). Apart from showing that messaging can effectively change thoughts about gambling and the gambling behavior itself, research has also suggested that the content of messages is important (Monaghan and Blaszczynski, 2010a,b; Auer and Griffiths, 2015b). The present authors take the view that the design of a feedback system for facilitating responsible gambling is paramount, and that HCI and PSD principles should be at the heart of such systems.

### The Present Study and Hypotheses

This study goes beyond previous research as it applies an experimental approach in a real-world online gambling setting. This is in contrast to Auer and Griffiths’ (2015b) study in which players voluntarily signed up for a service that provided them with personalized spending information. In this study, players were randomly assigned to different types of interventions in order to investigate the effects of personalized feedback, normative feedback, and non-personalized recommendations. Additionally, a control group was drawn that allows for the causal inference of the different types of interventions. The main research questions (RQs) were: (RQ1) Does personalized information given to gamblers reduce their gambling behavior? (RQ2) Do different types of personalized information (i.e., personalized feedback, normative feedback, a recommendation to gamble responsibly) given to gamblers reduce their gambling behavior in different ways? (RQ3) Are gamblers’ demographics and playing attributes associated with their gambling behavior in reaction to the various messaging interventions or do all gamblers react similarly to the specific interventions, regardless of the message attributes? It was hypothesized that compared to the control group, personalized feedback would impact positively on subsequent playing behavior as assessed by a reduction in time and money spent in the experimental groups (H1), and that the impact of personalized feedback and normative feedback would be larger compared to either a pure recommendation or no information at all (H2).

## Materials and Methods

### Participants

The participant sample drawn from the population were those that had played at least one game for money on the *Norsk Tipping* online platform (*Instaspill*) during April 2015. A total of 17,452 players were randomly selected from 69,631 players that fulfilled the selection criteria (see next section for Sampling procedure). Ten players had won more money than they wagered over the time period and were thus excluded from analysis leaving 17,442 participants. Of these, 12,261 were males (69.1%) and 5,481 were females (30.9%). All but 40 participants were Norwegian. The mean average age was 40.52 years (*SD* = 13.19). Approximately 29% of the customers were younger than 30 years, and 22% were aged over 50 years. There is no significant age difference between males and females. Participants had been playing with *Norsk Tipping* for a mean average of 94 months (7.9 years; *SD* = 38.31).

### Sampling

The participants only comprised players who had a net loss across all games in the past month before the study commenced (i.e., winners were excluded). Those who had self-excluded and/or taken a play break from gambling were also excluded from subsequent analysis. More specifically, the sample was drawn based on the amount lost across all games (online casino, sports betting, lottery, etc.) apart from scratchcards purchased oﬄine (as these data were not fully available during the study period). The amount of money lost by each player was computed by simply subtracting the amount wagered from the amount won. The overwhelming majority of players lost only small amounts of money. Therefore, in order to examine the impact of messaging on high intensity players, there was an oversampling of high intensity gamblers.

### Experimental Design

The study examined the effects of personalized messaging and manipulated the information given. **Table 1** contains the different permutations for the experimental design. The six groups were created based on three types of messaging intervention. These were:

**Table 1:** Experimental groups by type of feedback provided to gamblers.

	Personalized information	Recommendation	Normative feedback
Group 1	YES	NO	NO
Group 2	YES	YES	NO
Group 3	YES	YES	YES
Group 4	YES	NO	YES
Group 5	NO	YES	NO
Group 6	NO	NO	NO

1. Personalized information about the participant’s gambling behavior (in the form of numbers and illustrations).
2. A recommendation (in the form of written information about using responsible gaming tools offered by *Norsk Tipping*).
3. Normative feedback (in the form of numbers and illustrations displaying the gambling intensity of the average active player at *Norsk Tipping* compared to their own).

Group 5 was designed to evaluate the effectiveness of non-personalized, purely informative recommendation. Group 6 served as the control group as participants did not receive any information at all. The distribution of high and low intensity players created by the sampling procedure was the same across all six groups. Each group contained approximately 2,957 participants. In this 2 × 2 × 2 design, only six groups were examined (rather than the normal eight) because normative feedback without personalized feedback would not make any theoretical sense. This is also the case with providing normative feedback and recommendations without personalized feedback.

The study was planned over the course of 1 year and executed during May and June, 2015. During the course of the study, players (excluding the control group) received information about their losses over the past 6-month period and/or, recommendations about existing responsible gaming tools, and/or normative information. It was hypothesized that personalized behavioral feedback would help players to gamble more responsibly (as assessed using the TL, the amount of money wagered in Norwegian Krone [NOK], and the gross gaming revenue (GGR) [NOK] – see ‘Analysis’ section below for more detail).

All players that received information were told (in writing) that *Norsk Tipping* was trying out new services and that the operator would be very grateful if they could participate in the study. Players were told that they would be receiving information that would help them become more aware of their personal gambling expenditure. They were also informed that a research team had been asked to evaluate the effects of this service and that only anonymized data would be used for research purposes. Players had to press a ‘Next’ button to confirm that they agreed to participate and that their data would be used for research purposes. To not be included in the study, players were informed that they could simply close the message window. The demographic distribution was the same for all six groups (i.e., there were no significant differences across the six groups in terms of gender, age, and nationality). Of the 17,452 randomly selected players, 5,528 voluntarily participated in the study.

### Personalized Feedback

**FIGURE 1:**
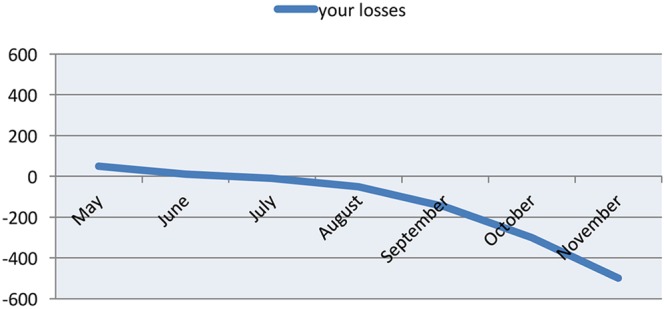
**Line chart containing personalized feedback of money lost (in NOK)**.

A simple personalized message was sent to players in Groups 1–4 that said: “How much do you think you spent on gambling recently? Our records show that you lost [X] NOK last month.” In addition, players were also presented with a line chart (see **Figure 1**) that contained the monthly values for their personal losses over the previous 6-month period. Players were also told that they could retrieve the information any time during the following month.

### Normative Feedback

**FIGURE 2:**
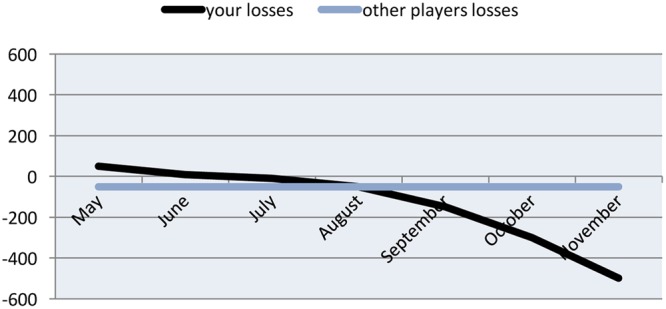
**Line chart containing personalized feedback of money lost (in NOK) compared to other players’ losses (i.e., normative feedback)**.

A simple message with normative feedback was sent to players in Groups 3 and 4 that said: *“It can be helpful to know about other peoples’ expenditures to evaluate your own spending. For this reason we would like to let you know that the average Norsk Tipping player loses about 400 NOK per month.”* The normative feedback about other players’ losses was provided after the personalized feedback. Additionally, a line chart (see **Figure 2**) displaying their own losses compared with those of other players was also provided.

### Recommendation

Groups 2, 3, and 5 received a helpful recommendation about responsible gambling tools and services that players could access via a hyperlink on the screen. Players could access tools provided by *Norsk Tipping* that helped players (i) manage their personal spending limits, (ii) activate a play break, (iii) take a diagnostic self-test about their gambling behavior, and (iv) see an overview of their recent spending. Players were also informed about the national gambling helpline if they wanted to speak to anyone about their gambling.

### Analysis

Assessing whether personalized feedback results in the desired behavioral change means that behavioral change has to be assessed via specific variables and via specific time periods. In the present study it was decided that gambling behavior 7 days prior to the intervention would be compared with gambling behavior 7 days after the intervention message was read. This is because changes over a shorter time period would most likely only be due to chance and changes over longer time periods would not be expected based on the type of feedback.

The three measures of behavior that were used to assess gambling behavior were TL, amount of money wagered, and GGR (i.e., net win/loss). The TL statistic was computed as ‘TL_after’ minus ‘TL_before’ divided by ‘TL_before.’ This statistic reflects the change in behavior 7 days after the message was read as a percentage of the behavior 7 days before the message was read. This procedure helps assess the individual change as independent from the intensity of play as much as possible. A negative value indicates a decrease in gambling behavior and a positive value indicates an increase in gambling behavior. A value of 0 means that no change in gambling behavior occurred at all. A value of say -0.5 means that the gambling behavior decreased by 50% compared to 7 days before. The statistic ranges from -1 to +infinity. A value of -1 means that the player did not engage in gambling 7 days after the message was read. A value of +10 means that the gambling behavior increased 1000% in the 7 days after the message was read compared to 7 days before the message was read. Prior to analysis, data cleaning was performed. More specifically, it appeared that a few customers (*n* = 28) had negative TL values that led to negative change metrics. Consequently, these 28 customers were removed from the analysis. An outlier procedure was also performed which removed the 1% highest values in the change metric as this was naturally highly skewed. The comparisons across the groups were performed via Mann–Whitney *U* Tests due to the skewed distribution of the metrics. Three statistical tests were carried out using chi-square analysis and using a Bonferroni correction, the significance level was determined to be 0.0167. The study was granted ethical approval by the research team’s University Ethics Committee.

## Results

### Theoretical Loss Analysis

**Table 2** displays the statistics of the TL change variable after data cleaning. The largest values (top 1%) were discarded from further analyses as these extreme outliers would influence the results heavily. Very large values occur if players were gambling little before the intervention and heavily after as a consequence of the quotient which is computed. It can be safely assumed that those players who spend very little and received feedback are not the main target group for such interventions. The arithmetic mean demonstrated that the average player increased their gambling by about 6%. However, it is evident that the distribution is highly skewed as the median is much smaller. Half of the players (50%) decreased their gambling by about 42%. The non-normal distribution is of course due to the fact that there is a lower limit at -1 and a much higher limit at 16 (i.e., the maximum increase was 1600%).

**Table 2:** Parametric (e.g., mean) and non-parametric (e.g., 1st quantile, median) statistics of the cleansed theoretical loss over a 7-day period.

Minimum	-1.00
1st Quantile	-0.87
Median	-0.42
Mean	0.06
3rd Quantile	0.15
Maximum	16.00

### Control Group Analysis

Players in the control group did not have a response date as there were no messages or recommendation sent to them. Players who received a message and read it had a specific response date on which they read the message. In order to do that they had to be online and be actively gambling. Given the missing response date in the control group, it is not easy to compare the computed change in gambling behavior 7 days after the message was read to a corresponding change in the control group. Simply looking at the gambling behavior in the same month as the other players received personalized messages is not valid as the responders in Groups 1–5 naturally showed some sort of activity and no such selection can be applied in the control group. Consequently, the level of monthly gambling across the responders is naturally higher than that of the control group. Therefore, an alternative procedure was chosen to analyze the data. In order to determine if the changes observed in Groups 1–5 were due to the message, it is important to find out how much players change on average when they do not receive a personalized message.

For each of the 2,958 control group members, every day on which they showed gambling activity was selected. This could be 1 day, a few days, or every day. For each and every control group player, a value between 0 and 30 with respect to active gambling days was calculated. For each and every player, and every active playing day, the gambling activity 7 days before that day and 7 days after that day was computed. Finally, the change statistic as used above was computed. Overall, it was assumed that all influences cancel each other out and that the average change statistic (across players and days) yields an average change in behavior.

### Message Effect Analysis

**Table 3** shows the distribution of the change in TL across five experimental groups and the control group. The control group statistics were computed across control group members and the respective days on which they were active. Examination of the median, mean, and maximum value clearly shows that the control group changed their gambling behavior less than the experimental groups. Whereas half of the players decreased their intensity by at least 42% (median) in the target groups, it was only 36% (median) in the control group. The average player (arithmetic mean) increased their play by 6% in the target group and 58% in the control group. Using a Mann–Whitney *U* Test, comparison of TLs after 7 days of receiving personalized messages between Groups 1 to 5 and the control group (Group 6) was significant (*X*^2^ = 32.208, df = 5, *p* < 0.0001). Thus, personalized feedback appears to have had a significant impact on player behavior compared to those that had no such feedback (see **Table 4**).

**Table 3:** Parametric (e.g., mean) and non-parametric (e.g., median, 1st quantile) statistics of the theoretical loss change variable by group mapping over a 7-day period).

	Target group	Control group
Minimum	-1	-1
1st Quantile	-0.87	-0.87
Median	-0.42	-0.36
Mean	0.06	0.58
3rd Quantile	0.15	0.37
Maximum	16.0	46.4

**Table 4:** Differences between the experimental groups (EGs) and control group (CG) 7 days after gamblers had received personalized messages about their gambling behavior

	CG	EGs	*X*^2^	d.f.	*p*
Theoretical loss	-36%	-42%	32.208	5	0.0001^∗^
Amount wagered	-34%	-43%	26.66	5	0.0001^∗^
Gross gaming	-48%	-58%	28.66	5	0.0001^∗^

*^∗^Bonferroni correction significance level = 0.0167.*

**FIGURE 3:**
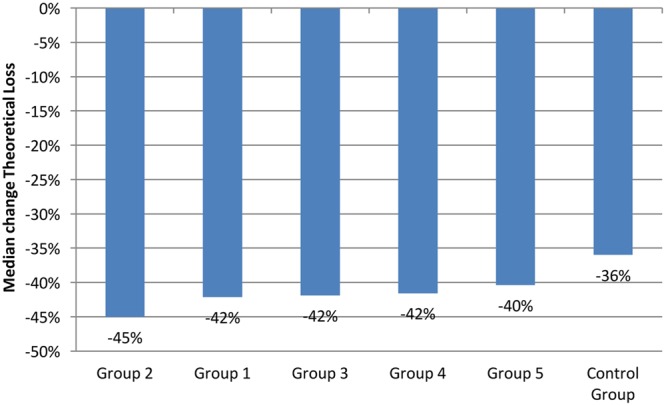
**Median 7-day change of theoretical loss variable by experimental group**.

**Figure 3** shows the change in TL across all six groups, 7 days after the message was read. Again, this shows that the control group (Group 6) decreased the least in relation to TL (-36%) and that Group 2 had the largest decrease in play in relation to TL (-45%).

**FIGURE 4:**
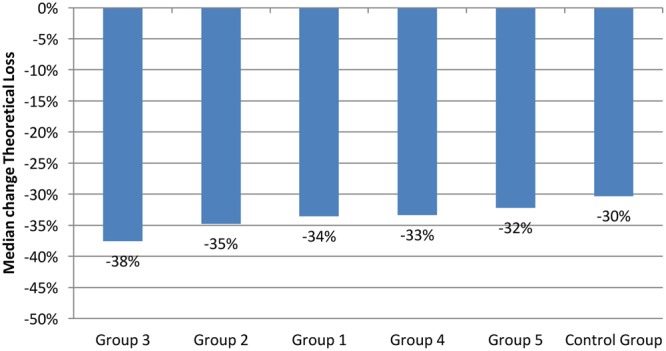
**Median 7-day change of theoretical loss variable by experimental group for 1st-week responders**.

Data also showed that the most gambling-intense players often deplete their financial resources during the 1st week of the month. During this time they reach their spending limits and can only resume playing at the site at the beginning of the next month. For that reason, a further analysis was carried out on those players who responded to the messaging during the 1st week of the study. **Figure 4** (using TL) shows the change in behavior 7 days after the message was read for players who responded during the 1st week of the study. It is again evident that using the TL, players who did not receive any message and players who only received purely informational content decreased their play less than players who received personalized feedback.

It is also important to examine whether the control group changed their gambling behavior with respect to the GGR and the amount of money wagered. The median change in amount wagered over a period of 7 days was -34%, and is a smaller decrease compared to the experimental Groups 1–5 (see **Table 4**), and was significant (*X*^2^ = 26.66, df = 5, *p* < 0.0001). The median change in GGR over a period of 7 days was -48%, and is a smaller decrease compared to the experimental Groups 1–5 (see **Table 4**), and was also significant (*X*^2^ = 28.66, df = 5, *p* < 0.0001).

### Sub-Group Analysis by Game Type and Demographics

The previous section demonstrated that players who received personalized feedback changed more than those who only received a recommendation and more than those who did not receive any kind of information. For this reason, further analysis investigated whether gamblers’ demographics and playing attributes were associated with their gambling behavior in reaction to the various messaging interventions. However, separating out all players into different clusters is difficult. In cluster analysis, statistical methods identify segments that are different from each other whereas the members of a single segment are similar to each other.

In this case, the goal was slightly different. If the study population was randomly divided into clusters, each cluster would be made up of some players who received personalized feedback, some players who received normative feedback, etc. However, the clustering should occur according to a specific goal. Here, members of a specific cluster that received personalized feedback should be different with respect to the behavioral change from members of that cluster who received normative feedback, etc. This means that an algorithm should uncover different clusters, whereas each cluster is characterized by a maximum variance across the five different interventions with respect to behavioral change.

In order to delineate groupings that have a unique profile with respect to the five experimental groups, a two-step approach was chosen. This consisted of a k-means statistical procedure and a genetic algorithm. Genetic algorithms are machine-learning procedures that imitate evolutionary processes. Parameters are randomly chosen and are optimized due to the performance of the algorithm that is measured via a fitness function. In this case, the fitness function was defined via the profiles of the clusters that the *k*-means produced. The more distinct the members of a cluster with respect to the five experimental interventions, the higher the corresponding fitness function. The open source statistical package ‘R’ was used to analyze the data and perform the genetic algorithm. (Details about the procedure and the parameters used in the genetic algorithm are available from the first author). Finally different types of players were identified based on this two-step approach (all distinct with respect to their characteristics as well as the reaction toward the different messaging interventions). This led to five distinct groups of player whose profile is displayed in **Table 5**. The following descriptions are an attempt to capture the main aspects of the five profiles of players in relation to game preference and GGR: Profile 1 – Female scratchcard players with average GGR; Profile 2 – Lottery players with a low GGR; Profile 3 – At-risk lottery players with higher GGR; Profile 4 – At-risk casino players with past self-exclusions and a high GGR; Profile 5 – At-risk male sports bettors who recently won with a below average GGR.

**Table 5:** Profile of five groups that reacted differently toward messaging interventions.

Player group	Profile 1	Profile 2	Profile 3	Profile 4	Profile 5	Average
Number of participants	348	1,940	1,208	1,010	404	4,910
GGR	430	293	673	738	370	494
Total amount wagered (NOK)	732	634	1,552	16,839	3,926	4,471
Number of playing days	8	7	13	15	15	11
Other games	2%	3%	14%	1%	2%	5%
Lottery	21%	67%	50%	7%	13%	43%
Online casino	75%	23%	23%	75%	11%	36%
Sports betting	1%	4%	7%	5%	56%	9%
Sport	0%	1%	3%	1%	15%	3%
Slots (land-based)	0%	2%	3%	12%	2%	4%
Casino (land-based)	2%	7%	6%	67%	5%	19%
Scratchcards	73%	16%	16%	2%	5%	16%
Bingo	0%	0%	1%	6%	1%	2%
Mean age (years)	39	37	48	46	41	42
Tenure	94	93	113	106	100	101
Gender (female)	47%	33%	30%	24%	5%	29%
Self-exclusion	8%	7%	16%	62%	23%	22%
*PlayScan* risk	49%	13%	75%	79%	64%	49%

The numbers in **Table 6** report the median 7-day change with respect to the theoretical loss in the five experimental groups for each player profile. For instance, players clustered in Profile 1 who were subject to Intervention 1 (personalized feedback only) reduced their gambling on average by 68% 7 days after they read the message. Players clustered in Profile 3 who were subject to Intervention 3 (personalized feedback, normative feedback, and a recommendation) reduced their gambling on average by 29% 7 days after they read the message. More specifically, the analysis demonstrated that:

**Table 6:** The effect of the different messaging interventions in each of the five experimental groups with respect to the change in theoretical loss.

	Profile 1	Profile 2	Profile 3	Profile 4	Profile 5
Intervention 1	-68%	-56%	-31%	-16%	-41%
Intervention 2	-87%	-53%	-35%	-27%	-21%
Intervention 3	-57%	-67%	-29%	-19%	18%
Intervention 4	0%	-57%	-35%	-22%	-17%
Intervention 5	-68%	-48%	-20%	-35%	-27%

• Profile 1: This group of players showed the largest decrease in play when presented with personalized feedback and a recommendation (i.e., a 68% reduction). The smallest decrease in play was when personalized and normative feedback were given together.
• Profile 2: This group of players showed the largest decrease in play when all three types of messaging were combined (personalized and normative feedback along with a recommendation). The smallest decrease in play was when they were given a recommendation only.
• Profile 3: This group of players showed the largest decrease in play when they were given personalized feedback and a recommendation or personalized and normative feedback. The smallest decrease in play was when they were given a recommendation only.
• Profile 4: This group of players showed the largest decrease in play when they were given a recommendation only. The smallest decrease in play was when they were given personalized feedback only.
• Profile 5: This group of players showed the largest decrease in play when they were given personalized feedback only. The combined intervention of personalized and normative feedback, and a recommendation appeared to increase play.

As shown in **Table 6**, in four of the five profiles, personalized feedback had a larger effect on gambling behavior (TL) than a sole recommendation. It was only in Profile 4 (highly involved at-risk casino players) where this did not hold true. Here, a sole recommendation had the largest effect on subsequent gambling behavior. The findings also suggest that although sports bettors benefit from personalized feedback, normative feedback plus a recommendation does not appear to make this group of players gamble less.

## Discussion

The present study examined whether the use of three types of information (i.e., personalized feedback, normative feedback, and a recommendation to gamble more responsibly) could enable players to gamble more responsibly as assessed using three measures of behavior, i.e., TL, amount of money wagered, and GGR. By manipulating the three forms of information, data from six different groups of players were analyzed. The first hypothesis was that compared to the control group, personalized feedback would impact positively on subsequent playing behavior as assessed by a reduction in time and money spent in the experimental groups in terms of TL, amount of money wagered and GGR. On the whole, empirical support for H1 was confirmed. The second hypothesis was that that the impact of personalized feedback and normative feedback would be larger compared to either a pure recommendation or no information at all. H2 was also empirically supported although the difference in reduction of gambling behavior by viewing a recommendation only (compared to personalized and normative feedback) did not quite reach statistical significance.

The first research question (RQ1) the present study attempted to answer was whether personalized feedback given to gamblers reduces their gambling behavior? The findings presented here suggest that it does and supports previous real world studies showing that personalized feedback about gambling behavior can help some players to decrease the amount of time and/or money they spend gambling (Auer et al., 2014; Auer and Griffiths, 2015a,b). However, almost all previously published research that has examined player feedback to date has been conducted in laboratory settings and has not looked beyond a single gambling session. The present study is a significant advance on those studies as it was carried out in the real world and for a period longer than within a single gambling session. Given this, the present study makes both a novel and original addition to the literature.

The second research question (RQ2) was to investigate whether different types of personalized feedback given to gamblers reduce their gambling behavior in different ways? Players that received both personalized feedback and a recommendation (Group 2) decreased gambling behavior the most although this was not statistically significant compared to the other four experimental groups. Those players given a recommendation only (Group 5) had the least change in their gambling behavior but again this did not quite reach statistical significance compared to the other four experimental groups. The other three groups (1, 3, and 4) showed equivalent decreases in gambling behavior. Findings suggest that the additional normative feedback received by Group 3 (who received all three types of message) did not have a more significant impact on gambling behavior than other groups. This may be due to a number of reasons. It could be due to the amount of information given (information overload – too many messages), or the fact that the types of gambler were diverse and that different messaging may impact more on different types of gambler. Furthermore, only one specific type of normative feedback was included in the present study (i.e., a comparison with the average player).

In relation to changes in GGR 7 days before and 7 days after the information was provided to players, gambling decreased more in Groups 1–4 (all of who received some kind of personalized feedback) compared to Group 5 (who received a recommendation only) supporting H1, although this did not quite reach statistical significance. This again tentatively supports the hypothesis that personalized feedback has a stronger effect in changing gambling behavior (as assessed using GGR) than pure informational content. In relation to changes in GGR 30 days before and 30 days after the information was provided, the findings were similar. Those groups that received some kind of personalized feedback (Groups 1–4) decreased their play (as assessed using GGR) after the message was provided. Those players that received a pure recommendation (Group 5) increased their play. Although across the groups as a whole the differences were not statistically significant, they are in line with H2.

In addition to TL and GGR, other studies (e.g., Broda et al., 2008; LaBrie et al., 2008; LaPlante et al., 2008, 2009; Nelson et al., 2008; Dragicevic et al., 2011; Braverman and Shaffer, 2012; Gray et al., 2012; Braverman et al., 2013) have frequently used the amount of money wagered as a proxy measure for gambling intensity. As with the findings above, the amount wagered by players 7 days after they had received personalized information was significantly less in the control group (supporting H1), and also less in players who only received a recommendation (supporting H2, although the finding was not statistically significant in the group that only received a recommendation compared to the other four experimental groups). After a period of 30 days, all groups tended to increase their play (as assessed using the amount wagered) after the information had been provided. Those that only received a recommendation (i.e., Group 5) showed the highest increase in amount wagered compared to the other four experimental group (supporting H2), although there was no significant difference across the five groups as a whole. Taken as a whole in relation to TL, GGR and amount wagered, the findings indicate that behavioral change is more intense in the days immediately after the information is provided to players, and also suggests that such information should be given more regularly.

The third research question (RQ3) investigated whether gamblers’ demographics and playing attributes are associated with their gambling behavior in reaction to the various messaging interventions or whether all gamblers react similarly to the specific interventions, regardless of the message attributes? To supplement and refine the analysis in relation to RQ3, different types of player were delineated using a *k*-means procedure and which led to five different player profiles based on gender, game-type, and GGR. These were: (i) female scratchcard players with average GGR, (ii) lottery players with a low GGR, (iii) at-risk lottery players with higher GGR, (iv) at-risk casino players with past exclusions and a high GGR, and (v) at-risk male sports bettors who recently won with a below average GGR. Results showed that in all profiles (bar the at-risk casino players), personalized feedback had a larger effect on gambling behavior than providing a sole recommendation. Providing a sole recommendation to at-risk casino players led to the most significant reduction in gambling behavior. Such findings, although arguably tentative, suggest that operators will need to use their data to more specifically target specific types of players. However, the findings presented here need to be replicated using other datasets from other operators before more general recommendations can be made to online gamblers as a whole.

Based on these findings, normative feedback might have worked better if it had been tailored to the type of player (e.g., giving sports bettors normative information about other sports bettors rather than gamblers in general) and therefore perceived as more relevant for the recipient. The comparison of TL data 30 days before compared to 30 days after the information was provided showed a slight increase in gambling behavior. However, the smallest increase in gambling was amongst those receiving personalized feedback and a recommendation (Group 2) and the largest increase in gambling behavior occur was among those receiving a recommendation only (i.e., Group 5).

### Limitations

Unlike the vast majority of studies carried out in the gambling studies field, the present study was a real world study using real online gamblers and carried out in real time. Furthermore, the sample size was relatively large and the dataset was robust. However, the study is not without its limitations. The players only comprised those that had gambled on the *Norsk Tipping* online platform during 1 month in 2015 and only evaluated the efficacy of viewing a single message on short-term behavior change in gambling (i.e., 1 week and 1 month after the message was viewed). Generalizations to other types of online gambler either in Norway or other countries cannot be assumed. Furthermore, players selectively retrieved the message and the information remained static, although the player behavior changed. It was also difficult to compare the players to the control group due to the fact that message retrieval was voluntary. Additionally it is not known if players read the message once or several times. Furthermore, the present study did not vary the display of the message content in terms of color or wording which may also have had a considerable impact on the message effect.

### Future Research

Based on the findings presented here, there are many possible avenues for further research. Such research could investigate (i) the use of personalized messages more than once (for instance, the showing of messages once every month), (ii) the effects of recent wins on playing behavior and to what extent personalized messaging helps or hinders further gambling behavior, (iii) secondary data analysis of behavior connected to the retrieval of personalized spending information, (iv) varying the message content (e.g., emotional vs. warning vs. informative), (v) personalized messaging that addresses specific types of behavior (binge gambling, high loss gambling, high win gambling, etc.), (vi) the effects of regular personalized feedback compared to one-time feedback (e.g., weekly vs. monthly feedback), and (vii) long-term gambling behavior of samples rather than just over a 1-month period.

## Conclusion

A number of conclusions based on the findings in the present study can be made. First, personalized behavioral feedback can enable behavioral change in gambling (based on data comparing those who received such messaging compared to those who did not). Second, additional normative feedback does not appear change behavior significantly more than personalized behavioral feedback. Third, patterns of gambling behavior associated with the effects of personalized messaging can be derived. Fourth, messages are most likely read during the 1st week after players have received them. Fifth, lottery players and female scratchcard players are more likely to read the message and act on messages than casino players. Sixth, lottery players (low and high spending) appear to benefit most from personalized feedback. Seventh, normative feedback does not appear to be beneficial for sports bettors, and high spending casino players do not appear to benefit from personalized feedback. In short, the data show that the effect of the three types of messaging (i.e., personalized feedback, normative feedback, and a recommendation) appears to depend upon players’ gambling habits as well as demographic and game-type factors. This is especially important in practical terms because operators will also need to take into account player attributes and behaviors when presenting different kinds of messages. The present study demonstrates one way in which operators could use the big data that they routinely collect to help inform and encourage responsible gambling among its clientele. The study also demonstrates the positive way in which academic researchers can collaborate in innovative research initiatives that ultimately help relevant stakeholder groups.

## Author Contributions

MA: Co-designed the study, analyzed the data, and contributed to writing of the paper. MG: Co-designed the study and contributed to writing the paper.

## Conflict of Interest Statement

The authors declare that the research was conducted in the absence of any commercial or financial relationships that could be construed as a potential conflict of interest.

Norsk Tipping provided access to the data and assisted in interpreting the results. However the writing of the paper, interpretation and conclusions reached were written in an independent capacity and not influenced.
